# The detection of faked identity using unexpected questions and mouse dynamics

**DOI:** 10.1371/journal.pone.0177851

**Published:** 2017-05-18

**Authors:** Merylin Monaro, Luciano Gamberini, Giuseppe Sartori

**Affiliations:** 1 PhD Program in Brain, Mind and Computer Science, University of Padova, Padova, Italy; 2 University of Padova, Human Inspired Technology Research Centre, Padova, Italy; 3 University of Padova, Department of General Psychology, Padova, Italy; Tianjin University, CHINA

## Abstract

The detection of faked identities is a major problem in security. Current memory-detection techniques cannot be used as they require prior knowledge of the respondent’s true identity. Here, we report a novel technique for detecting faked identities based on the use of unexpected questions that may be used to check the respondent identity without any prior autobiographical information. While truth-tellers respond automatically to unexpected questions, liars have to “build” and verify their responses. This lack of automaticity is reflected in the mouse movements used to record the responses as well as in the number of errors. Responses to unexpected questions are compared to responses to expected and control questions (i.e., questions to which a liar also must respond truthfully). Parameters that encode mouse movement were analyzed using machine learning classifiers and the results indicate that the mouse trajectories and errors on unexpected questions efficiently distinguish liars from truth-tellers. Furthermore, we showed that liars may be identified also when they are responding truthfully. Unexpected questions combined with the analysis of mouse movement may efficiently spot participants with faked identities without the need for any prior information on the examinee.

## Introduction

The use of faked identities is a very common issue. People can fake their personal information for a number of reasons. Faked autobiographical information is, for example, observed in sports, with players claiming to be younger than what they really are [1]. Social networks are plagued by faked profiles [2]. Faked personal identity is also a major issue in security [3]. In fact, a large number of terrorists are believed to be hidden among migrants from the Middle East entering Europe. Usually, migrants lack documents and their identity information is often based on self-declaration. Among migrants, it is believed that a high number of terrorists are giving false identities when entering borders. For example, one of the terrorists involved in the Brussels airport suicide bombing on March 22, 2016 was using the identity of a former Inter Milan football player [4]. In these cases, biometric identification tools (e.g., fingerprints) could not be applied as most of the suspects were previously unknown. Interestingly, detection techniques could be, in principle, applied.

From the beginning, starting with the pioneer work of Benussi [5], the identification of deceptive responses has mainly been based on the use of physiological measures [6]. More recently, reaction time (RT)-based techniques have been introduced. These are based on the response latencies to the presented stimulus of interest. There is wide consensus regarding the fact that deception is cognitively more complex than truth-telling and that this higher cognitive complexity is reflected in a number of indices of cognitive effort, including, for example, reaction times [7]. There is evidence that the process of inhibiting the truthful response, which is automatically activated, and substituting it with a deceptive response may be a complex cognitive task. However, in some instances, responding with a lie may be faster than truthfully responding [8]. In fact, distinct types of lies may differ in their cognitive complexity and may require different levels of cognitive effort. For example, the cognitive effort may be minimal when the subject is simply denying a fact that actually happened.

By contrast, it could be very high when fabricating complex lies, such as when Ulysses, the hero of *The Odyssey*, told Polypheme that his real name was “No-man.” This lie was intended to fool Polypheme but was also supposed to be easily spotted as a lie by Polypheme’s one-eyed companions.

RT-based memory detection has a number of advantages over alternative psychophysiological techniques, especially when a high number of subjects are under scrutiny. First, RTs are less sensitive to strong individual or environmental changes, such as in the case of physiological parameters. Secondly, this technique has the unparalleled feature that it may be applied using merely a computer and administered to a large number of examinees over the Web. Currently, two memory-detection techniques based on RTs that are used to present words or sentences may be adapted as tools for identity verification. The Concealed Information Test (CIT-RT) [9] and the autobiographical Implicit Association Test (aIAT) [10] are RT-based techniques that have undergone extensive scrutiny with satisfactory results [11].

The CIT-RT is a technique that consists of presenting the critical information within a series of very similar, noncritical sources of distractor information. For example, if the concealed knowledge about a murder weapon is under scrutiny, a knife (the known murder weapon) will be presented together with distractors that are also potential murder weapons (e.g., a gun, etc.). For the innocent subjects, the response is expected to be similar to all stimuli. By contrast, for the guilty subject (with guilty knowledge for the weapon), longer responses for the critical item are expected (e.g., the knife). When applied to verify whether the autobiographical information that the examinee claims corresponds to the true identity, the CIT efficiently succeeds in distinguishing the identities of liars and truth-tellers [11].

The aIAT is a memory-detection methodology that exploits consistency/inconsistency between sentences. It includes stimuli belonging to four categories: two of them are logical categories represented by sentences that are certainly true (e.g., “*I am in front of a computer*”) or certainly false (e.g., “*I am climbing a mountain*”) for the respondent and related to the moment of testing. The other two categories are represented by alternative versions of the autobiographical memory under investigation (e.g., “*I went to Paris for Christmas*” vs. “*I went to London for Christmas*”), with only one of the two being true. During the test, the examined subject performs a categorization task. The true autobiographical event is identified because it determines faster RTs when sharing the same motor response with certainly true sentences [12].

With regard to the average classification accuracy of RT-based lie-detection techniques, CIT [9] and aIAT [10] have similar accuracies to those of the experiments reported here (around 90%). Therefore, the technique reported here has similar accuracies to those of current RT-based lie-detection techniques. Nevertheless, the aIAT and CIT suffer from an important limitation: both of them require the true-identity information to be included in the test. The CIT-RT contrasts information about the true identity with information about the faked identity [11]. The aIAT is also built in such a way that, of the two contrasted memories, one should be true and one should be false [10]. If we build an aIAT only with the claimed (faked) identity, we will have two memories that are both false, and the test will not satisfy one of the basic constraints in the application of the procedure. This limitation of the available techniques is therefore a major issue for applications in real contexts, even if Meixer and Rosenfeld [13] carried out a step in this direction. In fact, in most investigative settings, the subject’s true identity is completely unknown to the examiner, who is interested in evaluating whether the claimed identity is true or not.

This paper could be considered a proof of concept, a representative example of types of problems that could not be addressed with current scientific-based lie-detection techniques (CIT and aIAT). Available techniques cannot be used when the critical information that is evaluated for veracity (in this case, the real identity of the respondent who is trying to hide his identity) is not available.

Here, we will present a new paradigm that overcomes the caveats of the available methods and may be used to identify whether personal information is true. Most importantly, we will show that a faked identity can be spotted in the absence of any information about the suspect’s true identity. Faked identities will be detected using unexpected questions combined with an analysis of mouse movements during the response in a binary classification task. We will show that the analysis of mouse dynamics efficiently detects whether the personal information that the examinee claims is true. In the experiments presented here, the participants do not respond by pressing YES/NO buttons using the keyboard, as in the RT-CIT or aIAT, but they are instead required to respond by clicking with a mouse virtual buttons appearing on the computer screen along with questions regarding their identities. The use of a mouse for recording responses has a number of advantages over the use of a keyboard. While the press of a button may permit only RTs to be recorded, mouse recording allows several indicators to be collected, including but not limited to RT (e.g., velocity, acceleration, and trajectory). The technique is also promising regarding resistance to countermeasures, as a high number of movement parameters seems, in principle, more difficult to control entirely via efficient, planned countermeasures to lie detection.

It has been shown that the analysis of mouse trajectories can capture cognitive complexity in stimulus processing when participants are required to deliver multiple-choice responses. This procedure has been applied to a large number of fields and has proved useful in highlighting cognitive complexity related to negative sentence verification [14], racial attitudes [15], perceptions [16], prospective memory [17], and lexical decisions [18]. Duran et al. presented a pioneering investigation on lie detection [19]. The authors recorded motor trajectories (the authors did not use a mouse to record the responses but rather a Nintendo Wii controller) while the subjects were engaged in an instructed lying task. During the task, the participants were required to respond truthfully or with lies to the presented sentences, as indexed by a visual cue. The analysis of motor trajectories led to interesting results. Instructed lies could be distinguished from truthful responses on several parameters, including the motor onset time, the overall time required for responding, the trajectory of the movement, and kinematic parameters, such as velocity and acceleration. Their experiment highlighted the fact that cognitive conflict induced by a lie affected the response trajectory but did not show directly its efficiency in classifying deceptive subjects from truth-tellers. In short, the technique that the authors investigated can be used to identify when a truth-teller lies but not when a liar lies, as their procedure compares, within the same truth-telling subject, truthful responses with lying responses.

Here, we will present the results of an experiment in which the trajectories of motor responses using the mouse were investigated while the participants were tested on questions regarding their identities. Two types of questions were asked: expected questions and unexpected questions [20]. Vrij and co-workers [21] pioneered the use of unexpected questions, and there is growing experimental support for the notion that, during investigative interviewing, deceptive subjects will be uncovered more easily using unexpected questions versus expected questions [22]. It has been shown that liars plan for possible interviews by rehearsing the questions they expect to be asked as well [23]. Liars give their planned responses to expected questions easily and quickly, but they need to fabricate plausible responses in the case of unexpected questions, and this yields an increase in the cognitive load. By contrast, truthful responses are not plagued by the side effects of the cognitive load as they are quite automatic and effortless for both expected and unexpected questions. Using the methodology of unexpected questions in investigative interviewing, Lancaster et al. [24] reported good classification rates for both truth-tellers (78%) and liars (83%). Lancaster et al. results [24] were observed by comparing the difference in the number of details reported when responding to expected and unexpected questions. In short, liars, with respect to truth-tellers, report many more details to the expected questions versus the unexpected questions, and lie detection can capitalize on this difference.

The experiment reported here consists of a binary classification task involving expected and unexpected questions about identity. Expected questions covered typical information as reported in documents, while unexpected questions covered information that was well known and automatically retrieved by the truth-teller but that should be “computed-on-the-spot” by liars. An example of an expected question would be one’s date of birth, and a corresponding unexpected question would be the zodiac corresponding to the date of birth. While truth-tellers easily verify questions involving the zodiac, liars do not have the zodiac immediately available, and they have to compute it for a correct verification. The uncertainty in responding to unexpected questions may lead to errors. Furthermore, we found that the trajectory mouse response, analyzed using kinematic parameters and other spatial and temporal parameters intended to capture the uncertainty in motor response, could be useful in detecting deception. Deception, therefore, is expected to reflect itself in the form of the trajectories.

## Methods

In an identity verification task, the liars are typically required to learn the autobiographical information of a new identity and to take the test responding as if that information were real for them. For example, Verschuere et al. [11] asked subjects to adopt a false identity and rehearse and recall it until their performance was errorless. Then, the liars were required to respond as if their new identity was the true one. Similarly, here we required the deceptive participants to learn a new identity. During the testing session, the participants were presented both expected and unexpected questions about their personal information. The expected questions included information about the false identity that was assigned to liars and rehearsed before the test until the subjects did not make any errors. The truth-tellers rehearsed their true identities. The expected questions were on the typical information reported on an identification (ID) card (e.g., name, surname, date of birth, place of birth). By contrast, the unexpected questions were identity-related questions to which the subjects were not prepared to respond. These unexpected questions were directly derived from the expected questions (e.g., the identity’s age and zodiac sign are derived from the date of birth; while questions about the date of birth are expected, questions about age and zodiac sign are unexpected). For example, if the subject was rehearsing the year of birth as it appeared on a fake ID card (e.g., 1988), a birth-related unexpected question was about the age (e.g., 38).

For a truthful responder, unexpected questions are supposed to elicit the correct response automatically. By contrast, an identity liar has to reconstruct the non-rehearsed unexpected information and verify it. Therefore, this process takes time before the response is emitted, which is reflected in longer RTs. In short, *“Unexpected questions will increase a liar’s cognitive load”* [20] and this is expected to reflect itself not only in the RT and in the number of errors but also in the mouse trajectories.

In the following, we will describe in detail the experiment structure and the measures collected. The ethics committee for psychological research of the University of Padova approved the experimental procedure.

### Participants

Forty Italian-speaking participants were recruited at the Department of Psychology of Padova University. The sample consisted of 17 males and 23 females. Their average age was 25 years (SD = 4.6), and their average education level was 17 years (SD = 1.8). All of the participants were right handed. These first 40 participants were used to develop the model that was later tested, for generalization, in a fresh new group of 20 Italian-speaking participants (10 liars and 10 truth-tellers). This second sample consisted of 9 males and 11 females. Their average age was 23 years (SD = 1.5), and their average education level was 17 years (SD = 0.83). Both groups of subjects provided informed consent before the experiment.

### Stimuli

Thirty-two sentences displayed in the upper part of the computer screen were presented to all of the participants. The squares representing the YES and NO responses were located in the upper left and upper right of the computer screen. Sixteen sentences required a YES response, and 16 required a NO response, for both the liars and the truth-tellers. The 32 experimental questions were preceded by 6 training questions (3 requiring a YES response and 3 requiring a NO response) on issues related to the identity not included in the experiment proper (e.g., “Is your weight 51 kg?”). Sentences that required a YES response belonged to the following categories:

- Expected questions: These included information that was rehearsed before the experiment, both for the truth-tellers and for the liars. The liars responded with personal information about fake identity profiles that the experimenter had assigned to them. The truth-tellers responded to questions regarding their true identities.
- Unexpected questions: The unexpected questions included information closely related to the false identities but not explicitly rehearsed before the experiment by either the truth-tellers or the liars. In this case, the liars responded to information related to the fake identities assigned to them, while the truth-tellers responded to the questions regarding their true identities.
- Control questions: Control questions were intermixed with the expected and unexpected questions. The control questions (*n* = 8; 4 requiring a YES response and 4 a NO response) included personal information to which the subjects had to respond truthfully because they could not be hidden to the examiner supervising the test. For example, “*Are you male*?” (for a male subject) required a YES response, whereas “*Are you a female*?” (for a male subject) required a NO response. Therefore, the control questions required truthful responses by both the liars and truth-tellers, even if they were related to identity.

For both the liars and the truth-tellers, half of the expected, unexpected, and control questions (*n* = 16) required YES responses. By contrast, 16 questions derived from the expected, unexpected, and control questions required NO responses as displayed in Table 1.

**Table 1:** Examples of expected questions, unexpected questions and control.

Topic	Profile that requires YES response by both liars and truth-tellers	Profile that requires NO response by both liars and truth-tellers
**Expected questions**		
Name	Is Giulia your name?	Is Caterina your name?
Surname	Is Rossi your last name?	Is Moretti your last name?
Year of birth	Were you born in 1995?	Were you born in 1991?
Month of birth	Were you born in January?	Were you born in April?
Town of residence	Do you live in Padova?	Do you live in Pisa?
Street of residence	Do you live in via dei Colli 9?	Do you live in via Piave 25?
**Unexpected questions**		
Age	Are you 21 years old?	Are you 25 years old?
Zodiac sign	Is Capricorn your zodiac sign?	Is Aries your zodiac sign?
Region of birth	Were you born in Lombardia?	Were you born in Campania?
Province of birth	Were you born in the province of Milano?	Were you born in the province of Napoli?
Region of residence	Do you live in Veneto?	Do you live in Toscana?
Capital town of the region of residence	Is Venezia the capital of the region where you live?	Is Firenze the capital of the region where you live?
**Control questions**		
Gender	Are you female?	Are you male?
Skin color	Is your skin white?	Is your skin brown?
Hair color	Do you have blond hair?	Do you have black hair?
Citizenship	Are you Italian?	Are you French?

The table reports examples of expected questions, unexpected questions and control questions related to truth or fake identity.

As can be seen in Table 2, the responses of the liars and truth-tellers differed only in the expected and unexpected YES responses. In fact, for the liars, the expected and unexpected questions regarding their faked identities were actually NO responses that, because they were lying, required YES responses. In other words, only the questions with expected and unexpected YES responses differentiated the two groups because the truth-tellers responded sincerely, while the liars cheated. For all of the other questions (control YES, control NO, expected NO, unexpected NO), both the liars and truth-tellers responded truthfully.

**Table 2:** Examples of expected, unexpected and control questions that require a YES or a NO response.

Sentence type	Question for liars	Question for truth-tellers
Expected YES (e.g., Town of residence)	Do you live in Padova?	Do you live in Padova?
Unexpected YES (e.g., Region of residence)	Do you live in Veneto?	Do you live in Veneto?
Control YES (e.g., Citizenship)	Are you Italian?	Are you Italian?
Expected NO (e.g., Town of residence)	Do you live in Firenze?	Do you live in Firenze?
Unexpected NO (e.g., Region of residence)	Do you live in Toscana?	Do you live in Toscana?
Control NO (e.g., Citizenship)	Are you French?	Are you French?

The table reports examples of expected questions, unexpected questions and control questions that require a YES or a NO response.

### Experimental procedure

The experiment was carried out using *MouseTracker* software [25]. Twenty participants answered truthfully, while the others were instructed to lie about their identities according to a false profile that was over-learned before starting the experiment, according to Verschuere et al. [11]. The 20 liars were instructed to learn a false identity from a faked Italian identity card, to which a photo of the subject was attached and that also reported false personal data. After the learning phase, the participants recalled the information they read on the ID card twice. Between the two recalls, they were required to perform some mental arithmetic as a distracting task. On the other hand, the truth-tellers also performed mental arithmetic and revised their real autobiographical data only once before starting the experiment. During the experimental task, the 6 expected questions, 6 unexpected questions, and 4 control questions described above were presented randomly intermixed. For each of the 16 questions that required a YES response, a similar question requiring a NO response was presented. Each participant responded to 32 questions plus 6 training questions that were not included in the analysis. Half of the time, the YES question appeared first, and during the other half, it appeared second. The participants initiated the presentation of each question by pressing a START button, which appeared in the center of the lower part of the computer screen. The response was given by pressing one of two response buttons appearing in the upper part of the computer screen, one in the upper-left corner and one in the upper-right corner.

### Data collection through mouse movement

For each response, the *MouseTracker* software recorded the mouse position from the starting point to the press of the button. Because the recorded trajectories had different lengths, each motor response was time normalized to permit the trials to be averaged and compared [25]. Using linear interpolation, the software calculated time normalization in 101 time frames. As a result, each trajectory had 101 time frames, and each time frame had corresponding X and Y coordinates. We identified the moment in time in which the two groups showed a maximum difference during the movement along the y-axis. These points of maximum difference in time were coded as Y18, Y29, and Y30 (the total time was preliminarily rescaled to 100 time frames according to the procedure that Freeman and Ambady [25] validated). Then, we calculated the velocity and acceleration in these time frames. *MouseTracker* software recorded by default also other spatial and temporal parameters. Here we report all the parameters preliminarily collected by the *MouseTracker* software and used to encode the mouse trajectory. The parameters collected from the motor responses to each of the questions were the following:

- Number of errors: the total number of errors in responding to the 32 questions
- Initiation time (IT): the time between the appearance of the question and the beginning of the mouse movement
- Reaction time (RT): the time between the appearance of the question and the virtual button-pressing performed with the mouse
- Maximum deviation (MD): the maximum perpendicular distance between the actual trajectory and the ideal trajectory (the line connecting the starting button with the expected response button)
- Area under the curve (AUC): the geometric area included between the actual trajectory and the ideal trajectory
- Maximum deviation time (MD-time): the time taken to reach the point of maximum deviation from the ideal trajectory
- x-flip: The total number of changes in directions of the mouse during the full trajectory on x-axis
- y-flip: The total number of changes in directions of the mouse during the full trajectory on y-axis
- X, Y coordinates over time (X_n_, Y_n_): the position of the mouse along the axis over time
- Velocity over time: the velocity of the mouse between two time frames
- Acceleration over time: the acceleration of the mouse movement between two time frames

The final list of candidate predictors included 13 variables, which mapped the various dimensions of the response: number of errors, Initiation Time (IT), Reaction Time (RT), Maximum Deviation (MD), Area Under the Curve (AUC), Maximum Deviation time (MD-time), x-flip, y-flip, Y30, Y29, Y18, Y30–Y29, and Y29–Y18. For each of the variables we computed the average value of the 32 responses for each participant.

### Correlation analysis and feature selection

A correlation analysis was conducted to highlight the independent variables that had the maximum correlation with the dependent variable (truth-tellers vs. liars) and minimum correlation across the independent variables [26]. We considered, for each feature, the mean value of all of the responses (both YES and NO responses) within each subject. The total of 13 independent variables were entered into the correlation analysis. The following features were selected on the basis of these criteria and later used as predictors to develop the machine learning (ML) classifiers: number of errors (r_pb_ = 0.68), AUC (r_pb_ = 0.53), MD-time (r_pb_ = 0.45), and Y29 (r_pb_ = 0.42) (r_pb_ is the value of the correlation between the dependent and independent variables).

## Analysis and results

In this section, the steps followed to analyze the data and the procedure used in developing the ML classifiers are reported.

Data and instructions to replicate the results are available as Supporting Information (see S1 and S2 Datasets, S1 and S2 Text).

### Analysis of trajectories

**Fig 1:**
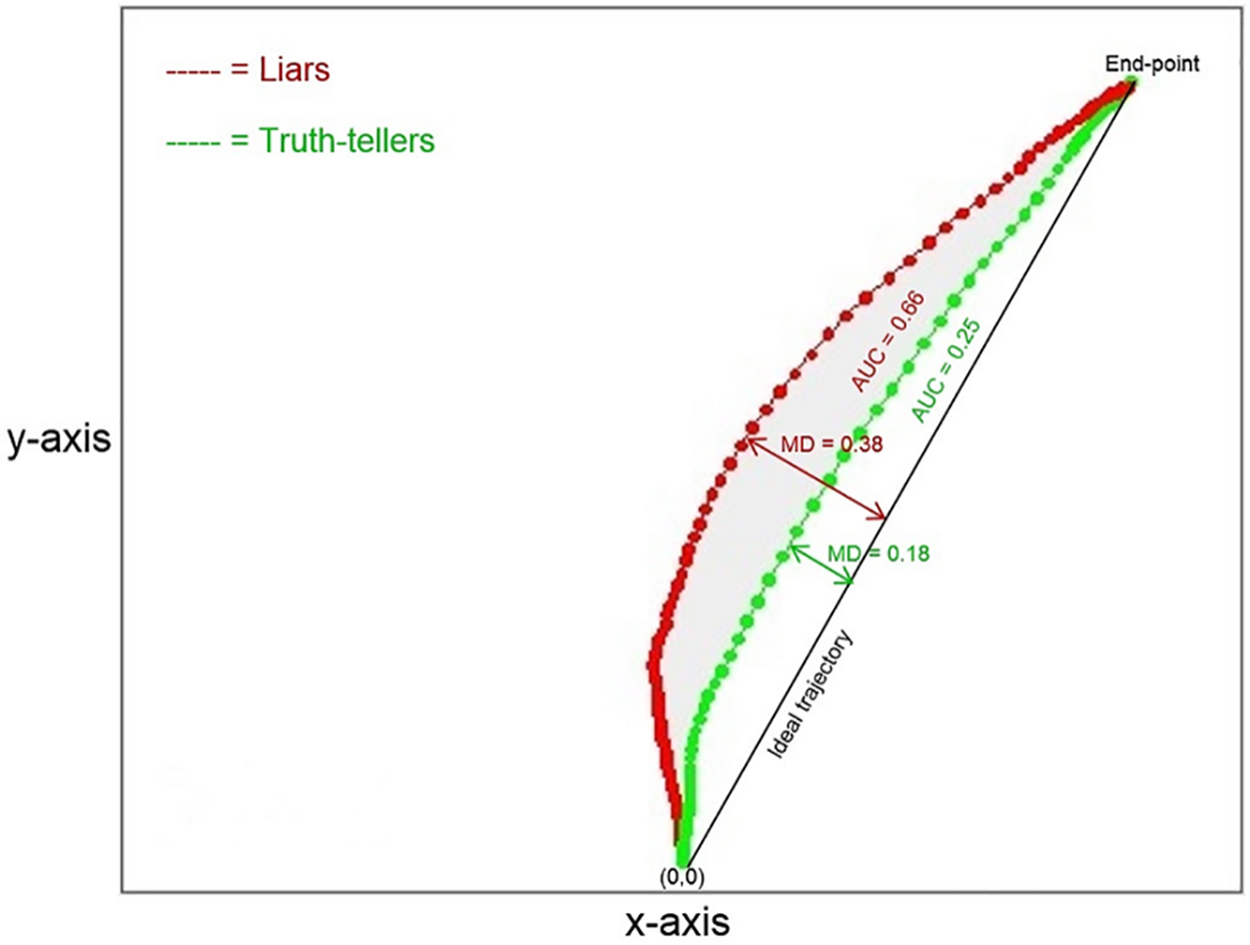
Average trajectories for liars and truth-tellers. The figure represents the average trajectories between the subjects, respectively for liars (in red) and for truth-tellers (in green), to the expected YES and unexpected YES questions. Expected and unexpected questions that require a YES response are those to which the liars lied. The values of the MD, AUC, x-flip, and y-flip parameters for the two groups are reported. The grey area represents the difference in the AUC parameter between the liars and truth-tellers.

The first analysis compared the responses of liars and truth-tellers by averaging across individual responses to both YES and NO responses. In Fig 1, the average trajectories for liars and truth-tellers responding YES to the expected and unexpected questions (the only questions to which the liars responded deceitfully) are represented. As can be noticed, the two experimental groups differed in both the AUC and MD parameters. The truth-tellers’ responses resulted in a more direct trajectory connecting the starting point with the correct response. By contrast, the liars initially deviated toward their default correct response and later changed their trajectory to press to false response button. Furthermore, liars spent more time moving on the y-axis in the initial phase of the response than the truth-tellers. The maximum difference between the two groups in mouse position along the y-axis was detected at time frame 29. Accordingly, the Y coordinate at this time frame (Y29) was also added as a predictor.

#### Prototypical trajectories of truth-tellers and liars

**Fig 2:**
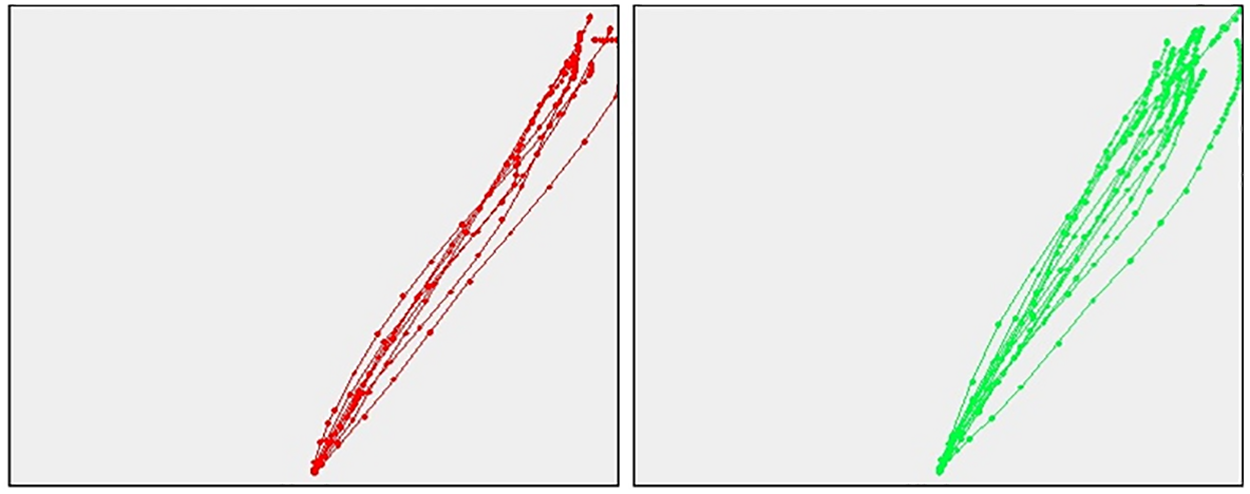
Prototypical trajectory of a truth-teller. Responses of a truth-teller (subject 3) to control (red) and unexpected questions (green). Trajectories refer to responses to single questions.

**Fig 3:**
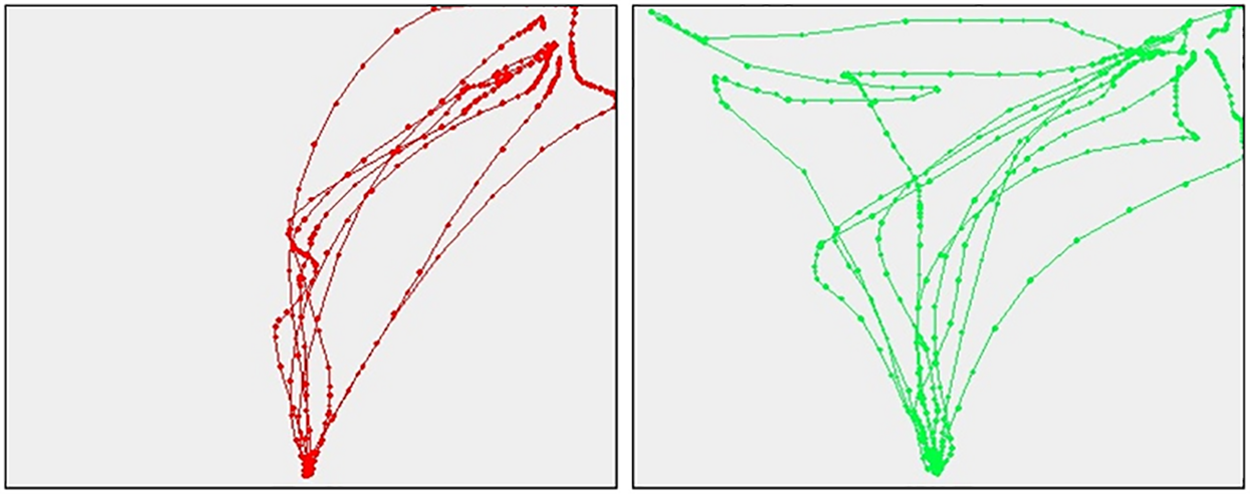
Prototypical trajectory of a liar. Responses of a liar (subject 2) to control (red) and unexpected questions (green).

Here we report examples of individual mouse trajectories in response to control questions and unexpected questions collected from a prototypical truth-teller (Fig 2) and a prototypical liar (Fig 3).

Trajectories refer to responses to single questions. Note that this liar is responding truthfully to control questions. Nonetheless, his response diverges from the direct trajectory that ideally characterizes a truthful response (see Fig 2). This generalization of the liar mindset when the liar is responding to questions that require truthful responses is discussed in the paper.

#### Disaggregation of responses to control, expected and unexpected questions

**Fig 4:**
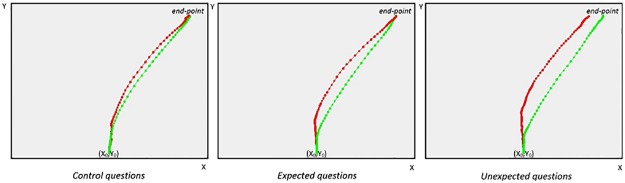
Trajectories for control, expected, and unexpected questions. Mouse trajectories for control questions (left), expected questions (center), and unexpected questions (right).

We analyzed the subjects’ performances separately for control, expected, and unexpected questions. In Fig 4, the trajectory for control, expected, and unexpected questions are reported (left to right). Trajectory of liars and truth-tellers in control questions are almost overlapping. The maximum difference in trajectory is observed again in response to unexpected questions.

#### Disaggregation of YES and NO responses

**Fig 5:**
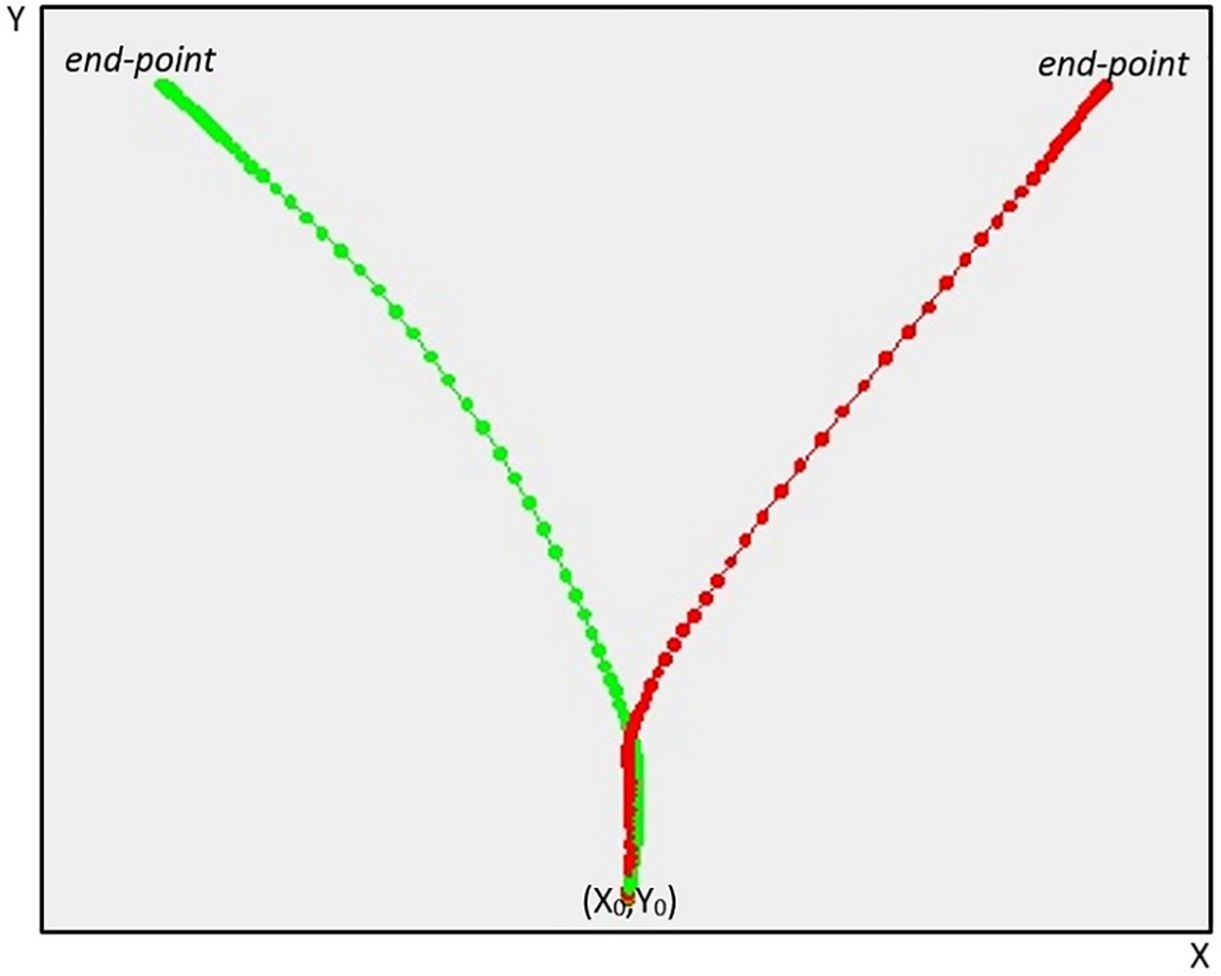
Trajectories for YES and NO responses. Responses to the left response button and to the right response button are reported separately here. The trajectory in the two types of responses did not differ.

We investigated whether there is a difference in the trajectory and in response time between the questions to which subjects responded by moving a mouse to the right (questions requiring a NO response) and questions to which subjects responded moving a mouse to the left (questions requiring a YES response). The *t*-tests on the whole sample were carried out in order to compare left and right responses. We did not find any statistically significant difference both for MD-time (*t* = 1.63; *p* = 0.1; Cohen’s *d* = 0.2; BF = 0.57) and Y29 (*t* = 0.1; *p* = 0.9; Cohen’s *d* = 0.01; BF = 0.17). For AUC, we obtained the following results: *t* = -2.09 and *p* = 0.04, but the Cohen’s *d* value showed a small effect size (*d* = -0.33), and the Bayes Factor approached (BF = 1.2). In Fig 5, trajectories of the left (green) and right (red) responses are reported. It can be noted that the two curves follow a very similar, albeit specular, trajectory.

### Descriptive statistics of independent variables

Feature selection isolated, from an original set of 13 predictors, 4 independent variables: errors, AUC, MD-time, and Y29. These were highly correlated with the group (truth-teller/liar). The following table (see Table 3) reports descriptive statistics as well as analysis of the difference between truth-tellers and liars as demonstrated by *t*-test, Cohen’s *d* and Bayes Factor.

**Table 3:** Descriptive statistics of the 13 independent variables.

	Mean (SD)		*t*-test		Cohen’s *d*	Bayes Factor
	Liars	Truth-tellers	*t*-value	*p*-value	*d*-value	Kass and Raftery interpretation
**Errors**	0.13 (±0.09)	0.01 (±0.02)	5.83	< 0.01	1.84 (large)	>150 (very strong)
**AUC**	0.60 (±0.38)	0.22 (±0.21)	3.09	< 0.01	1.23 (large)	70.41 (strong)
**MD time**	1264.33 (±377.78)	951.92 (±219.62)	3.19	< 0.01	1.01 (large)	13.52 (positive)
**Y29**	0.27 (±0.23)	0.11 (±0.08)	2.93	< 0.01	0.92 (large)	7.66 (positive)

Errors, AUC, MD-time, and Y29 were selected as the independent variables with maximum correlation with the group and minimal correlation among them. Average values are reported along with SD in parentheses and t-tests, Cohen’s d and Bayes Factor.

### Machine learning models

Several machine learning (ML) classifiers were tested using a 10-fold cross-validation procedure as implemented by WEKA [27]. We selected four classifiers that differ based on their assumptions: Random Forest [28], Logistic [29], Support Vector Machine (SVM) [30–31] and Logistic Model Tree (LMT) [32]. The 10-fold cross-validation was carried out as follows: the group of participants (40 subjects) was randomly subdivided in 10 subgroups of 4 subjects each. In each run, one of the 10 subsamples was retained as test set to evaluate the model and the remaining 9 were used as training data. The cross-validation process was then repeated 10 times so that each of the 10 subsets of participants were used exactly 1 time as validation set. The 10 results on the test set were then averaged to produce a single estimation of accuracy. The results are reported in Table 4. All of the classifiers reached an accuracy of around 90% or higher in classifying liars and truth-tellers. A minimum of 36/40 subjects were correctly classified. The Logistic classifier reached an accuracy of 95% (38/40 participants correctly classified). Comparable results have been obtained using a leave-one-out cross-validation (LOOCV) [33].

**Table 4:** Classification accuracy in the 10-fold cross-validation and in the validation sample.

ML classifier	Cross-validation	Validation sample	Misclassified
Random Forest	92.5%	95%	1/20–1 truth-teller
Logistic	95%	95%	1/20–1 truth-teller
SVM	90%	95%	1/20–1 liar
LMT	92.5%	95%	1/20–1 truth-teller

The accuracy in the 10-fold cross-validation is reported for four different ML classifiers: Random Forest, Logistic, Support Vector Machine (SVM), and Logistic Model Trees (LMT). Moreover, the table reports the accuracy percentages obtained in testing the previous models on a new sample of 20 participants and the number of misclassified subjects. Random Forest and LMT misclassified the same truth-teller, whereas Logistic misclassified a different one and SVM misclassified a liar.

As reported in Table 5, the classification models have both high specificity and high sensitivity. In fact, in the validation samples the classification errors are equally distributed in the two classes.

**Table 5:** Sensitivity and specificity of the classification models in the 10-fold cross-validation and in the validation sample.

	Cross-validation		Validation sample	
ML classifier	Specificity	Sensitivity	Specificity	Sensitivity
Random Forest	95%	95%	100%	95%
Logistic	97.5%	95%	100%	95%
SVM	100%	100%	95%	100%
LMT	92.5%	100%	100%	95%

Sensitivity and specificity in the 10-fold cross-validation and in the validation sample are reported for the four different ML classifiers: Random Forest, Logistic, Support Vector Machine (SVM), and Logistic Model Trees (LMT).

#### Model evaluation: Out-of-sample performance of 20 Italian participants

After the development of the ML classifiers described above, a further sample of 20 participants (10 liars and 10 truth-tellers) was collected and tested using the models previously developed based on the original 40 participants. This group of participants was a totally new group that had never been used before for the analysis or model building. This procedure is regarded as an optimal strategy to avoid overfitting (see Dwork et al. [34]). The classification accuracies on this new sample are reported in Table 4. It is worth noting that the classification accuracy remained stable, also across the classifiers, even in this validation sample.

#### Contribution of control, expected, and unexpected questions

To better understand the contribution of control, expected, and unexpected questions in the classification we ran three separate models for each type of question. Results indicate that the major contribution derives from unexpected questions (see Table 6). Classification accuracies using ML classifiers confirm that it is not possible to efficiently distinguish liars from truth-tellers solely based on control questions. The same is true also for expected questions although, in this case, the trajectories of the two groups seem to be more separated (see Fig 4). Using only unexpected questions, classification accuracy reaches its maximum with figures above 90%, also in the validation sample, confirming that the cognitive load of liars, due to unexpected questions, is at the origin of the difference between the two groups.

**Table 6:** Classification accuracy for control, expected, and unexpected questions.

Type of questions	Classifiers using	10-fold cross validation accuracy (*n* = 40)	Validation sample accuracy (*n* = 20)
**Control**	Random Forest	62.5%	30%
	Logistic	60%	60%
	SVM	55%	55%
	LMT	50%	60%
**Expected**	Random Forest	65%	55%
	Logistic	67.5%	50%
	SVM	65%	55%
	LMT	65%	55%
**Unexpected**	Random Forest	87.5%	100%
	Logistic	95%	95%
	SVM	92.5%	90%
	LMT	95%	90%

Classification accuracy using control, expected, and unexpected questions for each of the classifiers. Predictors were errors, AUC, MD-time, and Y29.

#### Relative weight of the predictors

We also investigated the relative weight of the predictors by eliminating the independent variables one by one and rerunning the classifiers. The results indicated that after eliminating the errors from the predictors, the classification accuracy dropped to around 75% for the cross-validation and around 70% for the test procedure (Random Forest: cross-validation = 70%, test = 65%; Logistic: cross-validation = 77.5%, test = 70%; SVM: cross-validation = 75%, test = 65%; LMT: cross-validation = 75%, test = 70%). The major contribution in prediction accuracy comes from revealing errors to unexpected questions with mouse dynamic features fine tuning an already good classification. This is clear if we consider that predictions based solely on errors yielded the following results: Random Forest: cross-validation = 77.5%, test = 100%; Logistic: cross-validation = 82.5%, test = 100%; SVM: cross-validation = 80%, test = 95%; LMT: cross-validation = 85%; Test = 100%. After dropping AUC from the predictors, the classification accuracy remained stable in the test set and fell to 90% during cross-validation (Random Forest: cross-validation = 90%, test = 95%; Logistic: cross-validation = 95%, test = 95%; SVM: cross-validation = 85%, test = 95%; LMT: cross-validation = 90%, test = 100%). Similar results were obtained when removing MD-time from the predictors (Random Forest: cross-validation = 90%, test = 95%; Logistic: cross-validation = 90%, test = 95%; SVM: cross-validation = 87.5%, test = 85%; LMT: cross-validation = 90%, test = 95%). Finally, after discharging Y29 from the predictors, the accuracy both in the training and the test sets decreased slightly (Random Forest: cross-validation = 92.5%, test = 95%; Logistic: cross-validation = 95%, test = 95%; SVM: cross-validation = 92.5%, test = 85%; LMT: cross-validation = 92.5%, test = 95%).

Briefly, the relative importance of the independent variables indicated that the total number of errors gave the major contribution in correctly distinguishing liars from truth-tellers, followed by the MD-time, the AUC, and the position of the mouse along the y-axis on the 29^th^ time frame.

#### Error analysis

The errors to control and expected questions are virtually absent in truth-tellers (see Table 7). Liars and truth-tellers made most of the errors to unexpected questions. The average liar makes 12.4 times the number of errors to unexpected questions with respect to truth-tellers.

**Table 7:** Analysis of errors.

Type of questions		Liars (*n* = 20)	Truth-tellers (*n* = 20)
**Control (*n* = 320 stimuli)**	Total number of errors / 160 stimuli	0/160	0/160
	Errors mean	0	0
	Errors SD	0	0
**Expected (*n* = 480 stimuli)**	Total number of errors / 240 stimuli for each group	2/240	2/240
	Errors mean	0.008	0.008
	Errors SD	0.091	0.091
**Unexpected all (*n* = 480 stimuli)**	Total number of errors / 240 stimuli	82/240	5/240
	Errors mean	0.341	0.020
	Errors SD	0.475	0.143
**Unexpected YES (*n* = 240 stimuli)**	Total number of errors / 120 stimuli	60/120	5/120
	Errors mean	0.5	0.042
	Errors SD	0.502	0.201
**Unexpected NO (*n* = 240 stimuli)**	Total number of errors / 120 stimuli	22/120	0/120
	Errors mean	0.183	0
	Errors SD	0.389	0

Errors to control, expected and unexpected questions for liars and truth-tellers. YES and NO responses are reported for unexpected questions.

Liars and truth-tellers make no errors to control questions and only a total 2/240 to expected questions. The difference between the two groups arises from unexpected questions with truth-tellers making a total 5/240 errors and liars 82/240. This indicates that for every error made by a truth-teller to unexpected questions liars make 16 errors. It is worth noting that liars make more errors to unexpected YES (60/120 where they lie) rather than unexpected NO (22/120 where they respond truthfully), *t* = - 4.59, *p*<0.01; Cohen’s *d* = 1.60; BF = 16.42.

#### German validation sample

To check whether the model can efficiently classify participants from different cultures, we tested 20 German subjects (10 liars and 10 truth-tellers) with good results. To address the effects of culture on the generalization of results, we tested a sample of 20 participants native speakers of German in Düsseldorf (10 truth-tellers and 10 liars; average age = 29.5 years; males = 9/20) with questions in German. Participants provided informed consent before the experiment. Results from this group were evaluated using the model originally trained on the 40 Italian participants. The classification accuracy was the following: Random Forest = 95%, Logistic = 100%, SVM = 90%, LMT = 95%. Errors analysis (see Table 8) indicates that the proportion of errors in liars and truth-tellers is comparable in the two groups (Italian *n* = 40 and German *n* = 20) with results for liars of *t* = -1.4, *p* = 0.17 (Cohen’s *d* = -0.49, BF = 0.64) and results for truth-tellers of *t* = 0.66, *p* = 0.52 (Cohen’s *d* = 0.28, BF = 0.43).

**Table 8:** Proportion of errors in liars and truth-tellers in the Italian and German samples.

Sample	Average number of errors	Total number of errors
Italian sample (n = 40)		
Liar	0.13/32	84/640
Truth-tellers	0.01/32	7/640
German sample (n = 20)		
Liar	0.17/32	55/320
Truth-tellers	0.006/32	2/320

The table reports the average and total number of errors in liars and truth-tellers of the the Italian and German samples.

### Can we detect liars also when they respond truthfully?

**Fig 6:**
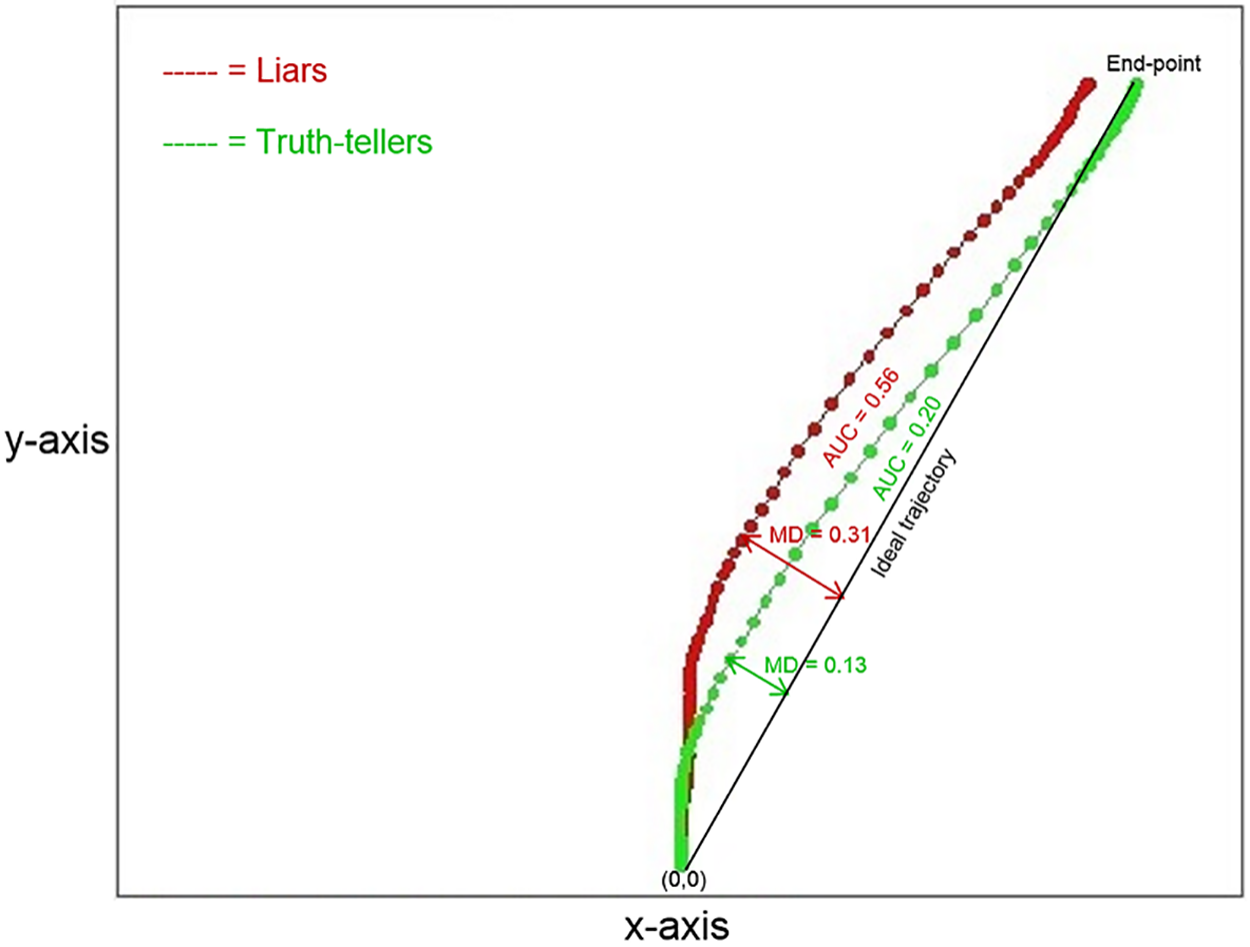
Trajectories for when the liars responded truthfully. In this figure, the average trajectories of the responses to questions where both liars (in red) and truth-tellers (in green) responded truthfully are reported.

The experimental design described in the manuscript requires that liars lie only when responding YES to expected and unexpected YES questions. In all of the other conditions (expected NO, unexpected NO, control YES, and control NO questions), the liars responded truthfully (see Table 2). An interesting question is whether the liars could also be spotted from their truthful responses. In the previous section, we compared the response trajectories of the two groups to expected and unexpected questions that required a YES response (see Fig 1). Here, we compared the trajectories of the two groups for the responses that required a NO response and for all of the control questions. The trajectories for when the liars responded truthfully are reported in Fig 6. Although the difference is reduced if compared with the responses for which the liars were lying, the differences with the truth-tellers are still detectable.

In order to evaluate whether the trajectories of the liars also differed from those of the truth-tellers when they were not lying, we compared the two experimental groups on the independent variables previously used in developing the classifiers. The results of the independent t-test, reported in Table 9, indicate that the liars’ response styles may be identified even when the liars were responding truthfully. The classifiers had the following accuracy rates in identifying liars and truth-tellers on the sole basis of responses to questions to which the liars responded truthfully: Random Forest = 77.5%, SVM = 80%, Logistics = 80% and LMT = 77.5%. All of the classifiers clearly were relatively accurate, even if below the classification accuracy based only on YES responses to expected and unexpected questions (which was in the range of 90–92%).

**Table 9:** Statistics for questions when the liars responded truthfully and for questions when the liars responded falsely.

Feature	Expected and unexpected YES questions	Expected and unexpected NO questions, and control questions
Number of errors	*t* = 6.06, *p* < 0.01, *d* = 1.91, BF > 150	*t* = 3.44, *p* < 0.01, *d* = 1.09, BF = 23.11
AUC	*t* = 3.46, *p* < 0.01, *d* = 1.09, BF = 24.46	*t* = 3.36, *p* < 0.01, *d* = 1.06, BF = 19.63
MD-time	*t* = 3.42, *p* < 0.01, *d* = 1.08, BF = 22.03	*t* = 2.65, *p* < 0.02, *d* = 0.83, BF = 4.37
Y29	*t* = 2.63, *p* < 0.02, *d* = 0.83, BF = 4.26	*t* = 2.98, *p* < 0.01, *d* = 0.94, BF = 8.51

The table reports the independent *t*-test analysis, Cohen’s *d* and Bayes Factor statistics when the liars responded falsely (expected and unexpected ES questions) and for questions when the liars responded truthfully (expected and unexpected NO questions, and control questions). The table reports *t*-values, *p*-values, Cohen’s *d* and Bayes Factor value for the four features that showed a high correlation with the dependent variable (liars vs truth-tellers).

Both statistical analysis and ML analysis have shown that the markers of lying extended to questions to which they responded truthfully. Even when responding truthfully, the liars could be identified, but with lower accuracy. From a cognitive point of view, what is interesting here is that, in the experimental design, the mind-set of the liars also extended its effects to questions when they were responding truthfully. To our knowledge, this pattern of results has never been reported before and could be an indication of the level of sensitivity of the technique of mouse-movement analysis.

## Discussion

To our knowledge, no techniques can accurately spot whether a subject’s ID is true or false without any information about the respondent’s true identity. In this paper, we report results on a new memory detection technique aimed at classifying whether an ID is true or faked when liars do not provide any personal information that is then included in the test itself.

The participants responded using a mouse to questions regarding ID that required a YES/NO response. Mouse dynamics provide a rich source of data, as compared to similar binary classification tasks based on response buttons. While the data collected through button presses are limited to recording latency between the question onset and the button press, mouse response permits several parameters to be collected, including reaction times but also initiation time, velocity, acceleration, and the mouse’s trajectory.

In order to develop a model that efficiently spots participants with faked identities, we tested the responders with questions that were expected and that liars over-learned in a preliminary learning phase (name, surname, date of birth, and place of birth). Together with expected questions targeting the ID document information, a set of unexpected questions related to the expected questions was also presented. Consider, for example, the place of birth. Expected questions that would appear on the ID card would be “Were you born in Pisa?” (requiring a YES response) or “Were you born in New York?” (requiring a NO response). Corresponding unexpected questions would be: “Is Florence the capital of your region of birth?” (requiring a YES response, given that Pisa, the place of birth, is in Tuscany, whose capital is Florence) and “Is Venice the capital of your region of birth?” (requiring a NO response, given that Pisa, the claimed place of birth, is in Tuscany, whose capital is Florence and not Venice). Another unexpected question related to the date of birth (derived from the date) was about zodiac. Truth-tellers are supposed to be able to retrieve the responses about their true zodiac more automatically than liars; therefore, their response is expected to be more rapid, with less errors, and characterized by a more direct mouse trajectory. In general, unexpected questions are supposed to be rapidly retrieved by truth-tellers while liars have to mentally “compute” the response from the original expected information [21].

The research reported here demonstrated that mouse dynamics analyzed using a ML model yielded a correct classification of liars and truth-tellers with more than 90% accuracy. This result was achieved by developing a set of classifiers with comparable performance in the accuracy range 90–95% (Random Forest, SVM, Logistics, and LTM). Another group was collected and tested (10 truth tellers and 10 liars) to validate the model’s generalization. In this group, the accuracy was confirmed to be comparable to that of the group used for developing the classifiers (95% = 19/20 participants correctly classified), showing that the high accuracy achieved in the model-building stage was not the result of overfitting.

Game theory is also a promising technique in deep learning. We did not evaluate whether more complex deep learning models based on game theory concepts [35–37] could outperform more than standard machine learning models that we used in this research, but it could be a future direction.

We conducted an analysis to identify the most important predictor, which was total errors followed by the MD-time, the AUC, and the position of the mouse along the y-axis on the 29^th^ time frame.

From a cognitive point of view, it is confirmed that unexpected questions may be used to uncover deception. The power of unexpected questions has been extensively examined in investigative interrogations [22]. Here, we extend the findings and confirm that unexpected questions may be embedded into an identity verification test to permit the identification of deceptive subjects with high accuracy. Liars find it hard to respond to unexpected questions quickly and without errors. Their uncertainty is captured by mouse dynamics, as their motor behavior diverges from the ideal truth-teller trajectory.

It is interesting to note that our experimental design requires liars to respond truthfully to a number of questions. The analysis performed on such truthful responses indicates that liars are still detectable, even if with a lower accuracy, when they are not lying. Rosenfeld et al. showed that truth telling liars could be identified using P300, similarly to what we report here [38]. It is important to note that liars are required to respond truthfully to all stimuli except to expected and unexpected questions, which, by contrast, require a lie. Therefore, they have to switch between lying and truth telling and this switch has a cost that reveals itself also when responding truthfully, as shown by Debey et al. [39]. This means that the liar mind-set reflects itself in the mouse dynamics and that lie detection could also be extended to responses to which they are not lying. It is as if being instructed to lie to some questions but not to others induces a greater cognitive load in liars, which is not only related to the deceptive responses but also to switching between responses that require a lie and responses that require the truth.

Unexpected questions require answers to be carefully crafted and this may be a limitation in online automatic usage of the technique. Further limitations of the present study include the fact that the procedure has been tested on participants of a single culture and generalization tested on participants belonging to a not so different culture (Germany). A further limit of the present research comes from the fact that the issue of faked ID detection does not permit a direct comparison with more validated lie detection techniques (e.g., CIT). Any comparison between techniques is therefore only indirect.

With all these limitations in mind, we think that the use of unexpected questions combined with the analysis of mouse dynamics seems to be a promising avenue for uncovering deceptive responses.

## Supporting information

S1 Text Data dictionary. (PDF)

S2 Text Instruction to replicate classification results. (PDF)

S1 Dataset Training set. (ZIP)

S2 Dataset Test set. (ZIP)
