# Supplementary material for: The detection of faked identity using unexpected questions and mouse dynamics
Source: PLoS One. 2017 May 18;12(5):e0177851. doi: 10.1371/journal.pone.0177851 (PMC5436828; doi:10.1371/journal.pone.0177851)
Supplement: S1 Text — (PDF) [file pone.0177851.s001.pdf]

S1 Text: Data Dictionary.

The data used by the authors to obtain classification results are available in .xlsx and .arff format. Particularly, two data files are available:

- **S1 Dataset.xlsx / S1 Dataset.arff** : it contains the data of the 40 participants that were used to run descriptive statistics and to develop the classification models.

- **S2 Dataset.xlsx / S2 Dataset.arff** : it contains the data of the 20 new participants that were used to test the classification models (model evaluation).

Each file contains the following attributes:

- Error: this is the average number of errors obtained by the subject on the 32 responses.
- AUC: this is the average AUC value obtained by the subject on the 32 responses.
- MD_time: this is the average MD-time value obtained by the subject on the 32 responses.
- Y_29: this is the average Y29 value obtained by the subject on the 32 responses.
- liar/truthteller: it is the class to predict. The label “liar” indicates that the subject was a liar participant, “truthteller” indicates that the subject was a truth-teller participant.

Abbreviations:

Area under the curve (AUC): the geometric area included between the actual trajectory and the ideal trajectory

Maximum deviation time (MD-time): the time taken to reach the point of maximum deviation from the ideal trajectory

Y29: the position of the mouse along the Y-axis at the time frame 29.
