# Supplementary material for: The detection of faked identity using unexpected questions and mouse dynamics
Source: PLoS One. 2017 May 18;12(5):e0177851. doi: 10.1371/journal.pone.0177851 (PMC5436828; doi:10.1371/journal.pone.0177851)
Supplement: S2 Text — (PDF) [file pone.0177851.s002.pdf]

S2 Text. Instructions to replicate classification results.

Machine Learning (ML) Models were implemented using WEKA software Version 3.8.

The software can be free downloaded at this link: http://www.cs.waikato.ac.nz/ml/weka/downloading.html . A complete documentation about WEKA software is available at this link: http://www.cs.waikato.ac.nz/ml/weka/ .

Classification results that are reported in the paper were obtained following these steps:

1) Open WEKA 3.8 and choose the *Explorer* application.
2) From the *Preprocess Panel* open the file "**S1 Dataset.arff**" (data from 40 participants). This file contains the data of the 4 features that the authors used in the paper to classify subjects as liars or truth-tellers: error, AUC, MD_time, Y_29. The variable named "liar/truthteller" is the class to predict.
3) Switch in *Classify Panel* and choose Cross-validation as *Test option* setting the number of folds to 10.
4) To replicate the paper results, from the *Classifier* list choose Random Forest, Logistic, SMO (SVM), LMT and run the models. The details on ML classifiers parameters are reported in **Annex 1**. The output of each classifier is reported in **Annex 2**.
5) To test the 4 model obtained by the 10-fold cross-validation on the validation sample (20 new participants), select Supplied test set as *Test option*. Upload the file "**S2 Dataset.arff**" as supplied test set. Then, to replicate the paper results on validation sample, choose Random Forest, Logistic, SMO (SVM), LMT from the *Classifier* list and run the models. The output of each classifier in the validation set is reported in **Annex 3**.

Annex 1 - Details on ML classifiers parameters

The analyses were carried out in WEKA (Version 3.8) using the default parameters. Note that there is no fine tuning of the parameters in order to increase the classification accuracy.

Random Forest:
weka.classifiers.trees.RandomForest -P 100 -I 100 -num-slots 1 -K 0 -M 1.0 -V 0.001 -S 1

- seed -- The random number seed to be used. = 1
- storeOutOfBagPredictions -- Whether to store the out-of-bag predictions. = FALSE
- numExecutionSlots -- The number of execution slots (threads) to use for constructing the ensemble. = 1
- bagSizePercent -- Size of each bag, as a percentage of the training set size. = 100
- numDecimalPlaces -- The number of decimal places to be used for the output of numbers in the model. = 2
- batchSize -- The preferred number of instances to process if batch prediction is being performed. More or fewer instances may be provided, but this gives implementations a chance to specify a preferred batch size. = 100
- printClassifiers -- Print the individual classifiers in the output. = FALSE
- numIterations -- The number of iterations to be performed. = 100
- debug -- If set to true, classifier may output additional info to the console. = FALSE
- outputOutOfBagComplexityStatistics -- Whether to output complexity-based statistics when out-of-bag evaluation is performed. = FALSE
- breakTiesRandomly -- Break ties randomly when several attributes look equally good. = FALSE
- doNotCheckCapabilities -- If set, classifier capabilities are not checked before classifier is built (Use with caution to reduce runtime). = FALSE
- maxDepth -- The maximum depth of the tree, 0 for unlimited. = 0
- calcOutOfBag -- Whether the out-of-bag error is calculated. = FALSE
- numFeatures -- Sets the number of randomly chosen attributes. If 0, int(log_2(#predictors) + 1) is used. = 0

Logistic:
weka.classifiers.functions.Logistic -R 1.0E-8 -M -1 -num-decimal-places 4

- numDecimalPlaces -- The number of decimal places to be used for the output of numbers in the model. = 4
- batchSize -- The preferred number of instances to process if batch prediction is being performed. More or fewer instances may be provided, but this gives implementations a chance to specify a preferred batch size. = 100
- debug -- Output debug information to the console. = FALSE
- ridge -- Set the Ridge value in the log-likelihood. = 1.0E-8
- useConjugateGradientDescent -- Use conjugate gradient descent rather than BFGS updates; faster for problems with many parameters. = FALSE
- maxIts -- Maximum number of iterations to perform. = -1
- doNotCheckCapabilities -- If set, classifier capabilities are not checked before classifier is built (Use with caution to reduce runtime). = FALSE

SMO:
weka.classifiers.functions.SMO -C 1.0 -L 0.001 -P 1.0E-12 -N 0 -V -1 -W 1 -K "weka.classifiers.functions.supportVector.PolyKernel -E 1.0 -C 250007" -calibrator "weka.classifiers.functions.Logistic -R 1.0E-8 -M -1 -num-decimal-places 4"

- buildCalibrationModels -- Whether to fit calibration models to the SVM's outputs (for proper probability estimates). = FALSE
- numFolds -- The number of folds for cross-validation used to generate training data for calibration models (-1 means use training data). = -1
- randomSeed -- Random number seed for the cross-validation. = 1
- c -- The complexity parameter C. = 1.0
- numDecimalPlaces -- The number of decimal places to be used for the output of numbers in the model. = 2
- batchSize -- The preferred number of instances to process if batch prediction is being performed. More or fewer instances may be provided, but this gives implementations a chance to specify a preferred batch size. = 100
- kernel -- The kernel to use. Polykernel –C 250007 -E 1.0
- checksTurnedOff -- Turns time-consuming checks off - use with caution. = FALSE
- debug -- If set to true, classifier may output additional info to the console. = FALSE
- filterType -- Determines how/if the data will be transformed. = Normalized training data
- toleranceParameter -- The tolerance parameter (shouldn't be changed). = 0.001
- calibrator -- The calibration method to use. = Logistic
- doNotCheckCapabilities -- If set, classifier capabilities are not checked before classifier is built (Use with caution to reduce runtime). = FALSE
- epsilon -- The epsilon for round-off error (shouldn't be changed). 1.0E-12

LMT:
weka.classifiers.trees.LMT -I -1 -M 15 -W 0.0
- splitOnResiduals -- Set splitting criterion based on the residuals of LogitBoost. There are two possible splitting criteria for LMT: the default is to use the C4.5 splitting criterion that uses information gain on the class variable. The other splitting criterion tries to improve the purity in the residuals produces when fitting the logistic regression functions. The choice of the splitting criterion does not usually affect classification accuracy much, but can produce different trees. = FALSE
- useAIC -- The AIC is used to determine when to stop LogitBoost iterations. The default is not to use AIC. = FALSE
- numDecimalPlaces -- The number of decimal places to be used for the output of coefficients. = 2
- batchSize -- The preferred number of instances to process if batch prediction is being performed. More or fewer instances may be provided, but this gives implementations a chance to specify a preferred batch size. = 100
- weightTrimBeta -- Set the beta value used for weight trimming in LogitBoost. Only instances carrying (1 - beta)% of the weight from previous iteration are used in the next iteration. Set to 0 for no weight trimming. The default value is 0. = 0.0
- doNotMakeSplitPointActualValue -- If true, the split point is not relocated to an actual data value. This can yield substantial speed-ups for large datasets with numeric attributes. = FALSE
- debug -- If set to true, classifier may output additional info to the console. = FALSE
- numBoostingIterations -- Set a fixed number of iterations for LogitBoost. If >= 0, this sets a fixed number of LogitBoost iterations that is used everywhere in the tree. If < 0, the number is cross-validated. = -1
- fastRegression -- Use heuristic that avoids cross-validating the number of Logit-Boost iterations at every node. When fitting the logistic regression functions at a node, LMT has to determine the number of LogitBoost iterations to run. Originally, this number was cross-validated at every node in the tree. To save time, this heuristic cross-validates the number only

once and then uses that number at every node in the tree. Usually this does not decrease accuracy but improves runtime considerably. = TRUE

- minNumInstances -- Set the minimum number of instances at which a node is considered for splitting. The default value is 15. = 15
- doNotCheckCapabilities -- If set, classifier capabilities are not checked before classifier is built (Use with caution to reduce runtime). = FALSE
- errorOnProbabilities -- Minimize error on probabilities instead of misclassification error when cross-validating the number of LogitBoost iterations. When set, the number of LogitBoost iterations is chosen that minimizes the root mean squared error instead of the misclassification error. = FALSE
- convertNominal -- Convert all nominal attributes to binary ones before building the tree. This means that all splits in the final tree will be binary. = FALSE

Annex 2 - Classifiers output in the 10-fold cross-validation

Random Forest output:

=== Run information ===

Scheme: weka.classifiers.trees.RandomForest -P 100 -I 100 -num-slots 1 -K 0 -M 1.0 -V 0.001 -S 1
Relation: training-weka.filters.unsupervised.attribute.Remove-R1
Instances: 40
Attributes: 5
error
AUC
MD_time
Y_29
liar/truthteller
Test mode: 10-fold cross-validation

=== Classifier model (full training set) ===

RandomForest

Bagging with 100 iterations and base learner

weka.classifiers.trees.RandomTree -K 0 -M 1.0 -V 0.001 -S 1 -do-not-check-capabilities

Time taken to build model: 0.02 seconds

=== Stratified cross-validation ===
=== Summary ===

Correctly Classified Instances	37	92.5 %
Incorrectly Classified Instances	3	7.5 %
Kappa statistic	0.85	
Mean absolute error	0.1878	
Root mean squared error	0.2805	
Relative absolute error	37.55 %	
Root relative squared error	56.1079 %	
Total Number of Instances	40	

=== Detailed Accuracy By Class ===

	TP Rate	FP Rate	Precision	Recall	F-Measure	MCC	ROC Area	PRC Area	Class
	0,900	0,050	0,947	0,900	0,923	0,851	0,956	0,967	liar
	0,950	0,100	0,905	0,950	0,927	0,851	0,956	0,949	truthteller
Weighted Avg.	0,925	0,075	0,926	0,925	0,925	0,851	0,956	0,958	

=== Confusion Matrix ===

```
  a  b   <-- classified as
 18  2 |  a = liar
  1 19 |  b = truthteller
```

Logistic output:

=== Run information ===

Scheme: weka.classifiers.functions.Logistic -R 1.0E-8 -M -1 -num-decimal-places 4
Relation: training-weka.filters.unsupervised.attribute.Remove-R1
Instances: 40
Attributes: 5
error
AUC
MD_time
Y_29
liar/truthteller
Test mode: 10-fold cross-validation

=== Classifier model (full training set) ===

Logistic Regression with ridge parameter of 1.0E-8
Coefficients...

Variable	Class<br>liar
error	6162.5451
AUC	320.7424
MD_time	0.8118
Y_29	-938.9489
Intercept	-1159.0654

Odds Ratios...

Variable	Class<br>liar
error	Infinity
AUC	1.9800286327824055E139
MD_time	2.2519
Y_29	0

Time taken to build model: 0 seconds

=== Stratified cross-validation ===
=== Summary ===

Correctly Classified Instances	38	95 %
Incorrectly Classified Instances	2	5 %
Kappa statistic	0.9	
Mean absolute error	0.0491	
Root mean squared error	0.2108	
Relative absolute error	9.8242 %	
Root relative squared error	42.1641 %	
Total Number of Instances	40	

=== Detailed Accuracy By Class ===

TP Rate FP Rate Precision Recall F-Measure MCC ROC Area PRC Area Class

	0,950	0,050	0,950	0,950	0,950	0,900	0,998	0,998	liar
	0,950	0,050	0,950	0,950	0,950	0,900	0,998	0,998	truthteller
Weighted Avg.	0,950	0,050	0,950	0,950	0,950	0,900	0,998	0,998	

=== Confusion Matrix ===

```
  a  b   <-- classified as
 19  1 |  a = liar
  1 19 |  b = truthteller
```

SMO output:

=== Run information ===

Scheme: weka.classifiers.functions.SMO -C 1.0 -L 0.001 -P 1.0E-12 -N 0 -V -1 -W 1 -K "weka.classifiers.functions.supportVector.PolyKernel -E 1.0 -C 250007" -calibrator "weka.classifiers.functions.Logistic -R 1.0E-8 -M -1 -num-decimal-places 4"
Relation: training-weka.filters.unsupervised.attribute.Remove-R1
Instances: 40
Attributes: 5
    error
    AUC
    MD_time
    Y_29
    liar/truthteller
Test mode: 10-fold cross-validation

=== Classifier model (full training set) ===

SMO

Kernel used:
  Linear Kernel: K(x,y) = <x,y>

Classifier for classes: liar, truthteller

BinarySMO

Machine linear: showing attribute weights, not support vectors.

```
       -2.5354 * (normalized) error
 +     -1.3845 * (normalized) AUC
 +     -1.3679 * (normalized) MD_time
 +     -0.7401 * (normalized) Y_29
 +      1.8417
```

Number of kernel evaluations: 202 (73.902% cached)

Time taken to build model: 0.02 seconds

=== Stratified cross-validation ===
=== Summary ===

```
Correctly Classified Instances          36               90      %
Incorrectly Classified Instances         4               10      %
Kappa statistic                          0.8
Mean absolute error                      0.1
Root mean squared error                  0.3162
Relative absolute error                 20      %
Root relative squared error             63.2456 %
```

Total Number of Instances 40

=== Detailed Accuracy By Class ===

	TP Rate	FP Rate	Precision	Recall	F-Measure	MCC	ROC Area	PRC Area	Class
	0,800	0,000	1,000	0,800	0,889	0,816	0,900	0,900	liar
	1,000	0,200	0,833	1,000	0,909	0,816	0,900	0,833	truthteller
Weighted Avg.	0,900	0,100	0,917	0,900	0,899	0,816	0,900	0,867	

=== Confusion Matrix ===

```
  a  b   <-- classified as
 16  4 |  a = liar
  0 20 |  b = truthteller
```

LMT output:

=== Run information ===

Scheme: weka.classifiers.trees.LMT -I -1 -M 15 -W 0.0

Relation: training-weka.filters.unsupervised.attribute.Remove-R1

Instances: 40

Attributes: 5

error

AUC

MD_time

Y_29

liar/truthteller

Test mode: 10-fold cross-validation

=== Classifier model (full training set) ===

Logistic model tree

: LM_1:5/5 (40)

Number of Leaves : 1

Size of the Tree : 1

LM_1:

Class 0 :

-2.83 +

[error] * 21.54 +

[AUC] * 1.41 +

[MD_time] * 0

Class 1 :

2.83 +

[error] * -21.54 +

[AUC] * -1.41 +

[MD_time] * -0

Time taken to build model: 0 seconds

=== Stratified cross-validation ===

=== Summary ===

Correctly Classified Instances	37	92.5 %
Incorrectly Classified Instances	3	7.5 %
Kappa statistic	0.85	
Mean absolute error	0.1534	
Root mean squared error	0.2816	
Relative absolute error	30.6795 %	
Root relative squared error	56.329 %	
Total Number of Instances	40	

=== Detailed Accuracy By Class ===

	TP Rate	FP Rate	Precision	Recall	F-Measure	MCC	ROC Area	PRC Area	Class
	0,850	0,000	1,000	0,850	0,919	0,860	0,925	0,955	liar
	1,000	0,150	0,870	1,000	0,930	0,860	0,925	0,890	truthteller
Weighted Avg.	0,925	0,075	0,935	0,925	0,925	0,860	0,925	0,922	

=== Confusion Matrix ===

```
  a  b   <-- classified as
 17  3 |  a = liar
  0 20 |  b = truthteller
```

Annex 3 - Classifiers output in the validation sample

Random Forest output:

=== Run information ===

Scheme: weka.classifiers.trees.RandomForest -P 100 -I 100 -num-slots 1 -K 0 -M 1.0 -V 0.001 -S 1
Relation: training-weka.filters.unsupervised.attribute.Remove-R1
Instances: 40
Attributes: 5
error
AUC
MD_time
Y_29
liar/truthteller
Test mode: user supplied test set: size unknown (reading incrementally)

=== Classifier model (full training set) ===

RandomForest

Bagging with 100 iterations and base learner

weka.classifiers.trees.RandomTree -K 0 -M 1.0 -V 0.001 -S 1 -do-not-check-capabilities

Time taken to build model: 0.03 seconds

=== Evaluation on test set ===

Time taken to test model on supplied test set: 0 seconds

=== Summary ===

Correctly Classified Instances	19	95 %
Incorrectly Classified Instances	1	5 %
Kappa statistic	0.9	
Mean absolute error	0.13	
Root mean squared error	0.208	
Relative absolute error	26 %	
Root relative squared error	41.5933 %	
Total Number of Instances	20	

=== Detailed Accuracy By Class ===

	TP Rate	FP Rate	Precision	Recall	F-Measure	MCC	ROC Area	PRC Area	Class
	1,000	0,100	0,909	1,000	0,952	0,905	1,000	1,000	liar
	0,900	0,000	1,000	0,900	0,947	0,905	1,000	1,000	truthteller
Weighted Avg.	0,950	0,050	0,955	0,950	0,950	0,905	1,000	1,000	

=== Confusion Matrix ===

```
  a  b   <-- classified as
 10  0 |  a = liar
  1  9 |  b = truthteller
```

Logistic output:

```
=== Run information ===

Scheme:       weka.classifiers.functions.Logistic -R 1.0E-8 -M -1 -num-decimal-places 4
Relation:     training-weka.filters.unsupervised.attribute.Remove-R1
Instances:    40
Attributes:   5
              error
              AUC
              MD_time
              Y_29
              liar/truthteller
Test mode:    user supplied test set:  size unknown (reading incrementally)

=== Classifier model (full training set) ===

Logistic Regression with ridge parameter of 1.0E-8
Coefficients...
                      Class
Variable               liar
===================================
error             6162.5451
AUC                320.7424
MD_time              0.8118
Y_29              -938.9489
Intercept        -1159.0654


Odds Ratios...
                      Class
Variable               liar
===================================
error              Infinity
AUC        1.9800286327824055E139
MD_time              2.2519
Y_29                      0


Time taken to build model: 0 seconds

=== Evaluation on test set ===

Time taken to test model on supplied test set: 0 seconds

=== Summary ===

Correctly Classified Instances          19               95      %
Incorrectly Classified Instances         1                5      %
Kappa statistic                          0.9
Mean absolute error                      0.05
Root mean squared error                  0.2236
```

Relative absolute error 10 %
Root relative squared error 44.7213 %
Total Number of Instances 20

=== Detailed Accuracy By Class ===

	TP Rate	FP Rate	Precision	Recall	F-Measure	MCC	ROC Area	PRC Area	Class
	1,000	0,100	0,909	1,000	0,952	0,905	1,000	1,000	liar
	0,900	0,000	1,000	0,900	0,947	0,905	1,000	1,000	truthteller
Weighted Avg.	0,950	0,050	0,955	0,950	0,950	0,905	1,000	1,000	

=== Confusion Matrix ===

```
  a  b   <-- classified as
 10  0 |  a = liar
  1  9 |  b = truthteller
```

SMO output:

=== Run information ===

Scheme: weka.classifiers.functions.SMO -C 1.0 -L 0.001 -P 1.0E-12 -N 0 -V -1 -W 1 -K "weka.classifiers.functions.supportVector.PolyKernel -E 1.0 -C 250007" -calibrator "weka.classifiers.functions.Logistic -R 1.0E-8 -M -1 -num-decimal-places 4"
Relation: training-weka.filters.unsupervised.attribute.Remove-R1
Instances: 40
Attributes: 5
error
AUC
MD_time
Y_29
liar/truthteller
Test mode: user supplied test set: size unknown (reading incrementally)

=== Classifier model (full training set) ===

SMO

Kernel used:
Linear Kernel: K(x,y) = <x,y>

Classifier for classes: liar, truthteller

BinarySMO

Machine linear: showing attribute weights, not support vectors.

```
       -2.5354 * (normalized) error
 +     -1.3845 * (normalized) AUC
 +     -1.3679 * (normalized) MD_time
 +     -0.7401 * (normalized) Y_29
 +      1.8417
```

Number of kernel evaluations: 202 (73.902% cached)

Time taken to build model: 0.02 seconds

=== Evaluation on test set ===

Time taken to test model on supplied test set: 0 seconds

=== Summary ===

Correctly Classified Instances	19	95	%
Incorrectly Classified Instances	1	5	%
Kappa statistic	0.9		
Mean absolute error	0.05		

Root mean squared error 0.2236
Relative absolute error 10 %
Root relative squared error 44.7214 %
Total Number of Instances 20

=== Detailed Accuracy By Class ===

	TP Rate	FP Rate	Precision	Recall	F-Measure	MCC	ROC Area	PRC Area	Class
	0,900	0,000	1,000	0,900	0,947	0,905	0,950	0,950	liar
	1,000	0,100	0,909	1,000	0,952	0,905	0,950	0,909	truthteller
Weighted Avg.	0,950	0,050	0,955	0,950	0,950	0,905	0,950	0,930	

=== Confusion Matrix ===

a b <-- classified as
9 1 | a = liar
0 10 | b = truthteller

LMT output:

=== Run information ===

Scheme: weka.classifiers.trees.LMT -I -1 -M 15 -W 0.0

Relation: training-weka.filters.unsupervised.attribute.Remove-R1

Instances: 40

Attributes: 5

error

AUC

MD_time

Y_29

liar/truthteller

Test mode: user supplied test set: size unknown (reading incrementally)

=== Classifier model (full training set) ===

Logistic model tree

: LM_1:5/5 (40)

Number of Leaves : 1

Size of the Tree : 1

LM_1:

Class 0 :

-2.83 +

[error] * 21.54 +

[AUC] * 1.41 +

[MD_time] * 0

Class 1 :

2.83 +

[error] * -21.54 +

[AUC] * -1.41 +

[MD_time] * -0

Time taken to build model: 0.02 seconds

=== Evaluation on test set ===

Time taken to test model on supplied test set: 0 seconds

=== Summary ===

Correctly Classified Instances	19	95	%
Incorrectly Classified Instances	1	5	%
Kappa statistic	0.9		
Mean absolute error	0.1151		
Root mean squared error	0.1911		
Relative absolute error	23.0248 %		
Root relative squared error	38.214 %		
Total Number of Instances	20		

=== Detailed Accuracy By Class ===

	TP Rate	FP Rate	Precision	Recall	F-Measure	MCC	ROC Area	PRC Area	Class
	1,000	0,100	0,909	1,000	0,952	0,905	1,000	1,000	liar
	0,900	0,000	1,000	0,900	0,947	0,905	1,000	1,000	truthteller
Weighted Avg.	0,950	0,050	0,955	0,950	0,950	0,905	1,000	1,000	

=== Confusion Matrix ===

```
  a  b   <-- classified as
 10  0 |  a = liar
```

1 9 | b = truthteller
